# Clinico-pathological assessment of surgically removed abdominal wall endometriomas following cesarean section

**DOI:** 10.1016/j.amsu.2021.01.029

**Published:** 2021-01-21

**Authors:** Abdulkarim Hasan, Abdou Deyab, Khaled Monazea, Abdoh Salem, Zahraa Futooh, Mahmoud A. Mostafa, Ahmed Youssef, Mohamed Nasr, Nasser Omar, Ali A. Rabaan, Doha M. Taie

**Affiliations:** a Department of Pathology, Faculty of Medicine, Al-Azhar University, Cairo, Egypt; b Department of Obstetrics and Gynecology, Faculty of Medicine, Al-Azhar University, Cairo, Egypt; c Department of General Surgery, Faculty of Medicine, Al-Azhar University, Assiut Branch, Egypt; d Department of General Surgery, Faculty of Medicine, Al-Azhar University, Cairo, Egypt; e Department of General Surgery, Faculty of Medicine for Girls, Al-Azhar University, Cairo, Egypt; f Department of Internal Medicine and Cardiology, King Fahad Hospital, Albaha, Saudi Arabia; g Department of Surgical Oncology, Faculty of Medicine, Al-Azhar University, Cairo, Egypt; h Department of Histology, Faculty of Medicine, Al-Azhar University, Cairo, Egypt; i Molecular Diagnostic Labortaory, Johns Hopkins Aramco Healthcare, Dhahran, Saudi Arabia; j Department of Pathology, National Liver Institute, Menoufia University, Shebin El Kom, Egypt

**Keywords:** Abdominal wall, Cesarean section, Endometriosis surgery, Pathology

## Abstract

**Background:**

Over the past few decades, the rate of Cesarean Section (CS) delivery has been rising rapidly and the prevalence of CS-associated complications including Abdominal Wall Endometriomas (AWE) increases with each additional operation. The aim of this study was to evaluate the clinical characteristics, histopathological diagnostic role and surgical management of post-CS AWE through a retrospective case review.

**Methods:**

We calculated the incidence of AWE and reviewed all the patients underwent surgical removal of Post-CS AWE during the period of 2012–2018 who were diagnosed, treated and followed up for 2–8 years at our tertiary hospital.

**Results:**

Thirty women with AWE were included. The main symptom in 2/3 of cases was cyclic pain and 4 cases (13.3%) had no symptoms. The mean interval between prior CS and appearance of symptoms was 55.2 months and the mean size of the excised mass was 42 mm. Free surgical margin was less than 9 mm in 9 patients (30%) but no recurrence was recorded among all the studied patients. Pre-operative FNAC diagnosis was performed for only 3 patients (10%) which helped in excluding other potential pathologies. The clinical–pathological agreement value for detection of the nature of the abdominal wall mass was 93.4%.

**Conclusions:**

Patients with suspected AWE should undergo preoperative cytological biopsy to exclude alternative diagnosis. Wide surgical excision with margin of less than 1 cm could be accepted especially in case of weak abdominal wall. More studies on the post-CS complications; risks, prevention, early detection and proper management should be encouraged.

## Highlights

• The incidence of post-CS abdominal wall endometriosis (AWE) in our hospital is 0.21%.
• Surgical excision of AWE with free margins of less than 1 cm could be acceptable.
• Pathology examination is crucial for AWE after excision but not a preoperative tool.

## 1 Introduction

Endometriosis is a benign inflammatory lesion characterized by presence of endometrial tissue outside the uterine cavity. When the lesion forms a circumscribed mass or blood-filled cystic lesion, hence designated as an endometrioma [1]. Abdominal wall endometrioma (AWE) is a rare aftermath of gynecologic operations mainly caesarean section (CS), myomectomy and abdominal hysterectomy causing intense pain and discomfort to the patient [2]. Malignant transformation of AWE is extremely rare but still a possibility; transformation to clear cell carcinoma and endometrioid carcinoma has been reported in several studies exhibiting aggressive prognoses [[3], [4], [5]]. CS scars are the most common site of abdominal or pelvic wall endometriomas, with an approximately estimated incidence of 0.03%–0.4% [6]. The increasing rate of CSs has raised concerns about the complications of the procedure. This rate has been increasing steadily in Egypt and has reached an alarming level in recent years. The proportion of documented CSs in Egypt in 2014 quadrupled the maximum recommended threshold by the World Health Organization (WHO) [7]. Direct inoculation of the endometrial cells into abdominal wall fascia or subcutaneous tissue during surgery is considered the main cause of scar endometrioses which subsequently stimulated by estrogen and the produced estrogen by the endometriotic lesions has a substantial role in lesions' development and progression [8]. Fine needle aspiration cytology (FNAC) before excision runs the risk of needle track inoculation and implantation of the endometriotic lesion but histopathological examination of the resected mass is a good confirmatory tool [2]. Several treatment options for AWE have been suggested including surgical and non-surgical (pharmacological) treatment, but the definitive and the gold standard treatment is wide surgical excision (WSE) with 5–10 mm free margins to prevent recurrence [9]. Awareness of this entity within the abdominal wall can help the surgeons to make early and accurate diagnosis and deliver prompt surgical intervention [2].

The main objective of this study was to assess the main clinicopathological characteristics and therapeutic options in AWE to make recommendations to improve diagnosis, treatment and prognosis.

## 2 Methods

Medical files and pathology reports between January 2012 and November 2018 for female patients underwent surgical excision of abdominal wall endometriomas by a general surgeon or a gynecologist at Al-Azhar university hospital were reviewed and evaluated with regard to patient age, history of CS operation, main patient complaint, blood Hemoglobin level, clinical suspicion, mass size, surgical margin status, post operative complications and recurrence. We collected any missed clinical data from the hospital registry, surgeon in concern or from the pathology request which usually contains the demographic data [10].

The inclusion criteria include; female patients with history of cesarean section operation at any time who came back with scar site mass during the time period of the study which proved endometriosis with histopathology. The exclusion criteria include; abdominal wall endometriosis with previous history of hysterectomy or any other surgical procedure and patients with abdominal wall endometriosis without complete follow up at our hospitals (2–8 years follow up).

This retrospective study included 30 patients. All masses were proved to be post-CS endometriosis by wide excision biopsy under spinal or general anesthesia with mask in OB/Gyn department or general surgery department and were confirmed by histopathology. Three cases were preoperative diagnosed using FNAC and the smears were reviewed by the histologist and pathologist authors. Hematoxylin and Eosin (H&E) stained slides for all the excision biopsies of the studied cases were reviewed by the three pathologists and the histologist who also reviewed the staining and processing.

Also the rate of AWE among the previously experienced females with cesarean section operations was calculated. Ethical approval was obtained from the local research ethics committee. This study has been reported in line with the STROCSS criteria [11].

**Statistical analysis:** Thirty lesions were evaluated and correlated with the clinical and pathological features then statistically analyzed using Excel program (Excel, Microsoft Corporation, Redmond, USA). The Interrater reliability between clinical and pathological final diagnosis was statistically assessed by kappa test. It is suggested by Cohen that the Kappa value be interpreted as follows: the value ≤ 0 is indicating no agreement, 0.01–0.20 is none to slight, 0.21–0.40 means fair, 0.41–0.60 is moderate, 0.61–0.80 reflects substantial, and 0.81–1.00 is almost perfect agreement [12].

## 3 Results

AWE in the studied cases of female patients with previous cesarean delivery during the period of 2012–2018 showed an incidence of 0.21% (30/14100).

The mean patient age was 35 ± 7.7 years ranged between the ages of 25 and 55 years, and the mean abdominal wall mass size was 42 ± 7 mm (Table 1). Nineteen patients (63.3%) had cyclic pain from the abdominal wall as a main complaint, seven (23.3%) patients was complaining of mass and discomfort and four (13.4%) did not experience any complaints but accidently discovered by the physicians during abdominal examination. Of 30 patients with complete follow up at our hospital and the available medical records, the interval from the most recent CS operation to abdominal wall mass first detection was 55.2 ± 25.3 months (range 12–118 month). Mean Hemoglobin level was 10.9 ± 2 (range 7.5–14.7 g per deciliter). Minor complications were recorded including wound infection in 5 patients and bleeding in other 4 patients, but the only one significant complication was incisional hernia in one case which was operated one year ago for an endometrioma of 5 cm diameter. A surgical excision with polypropylene mesh closure was performed for two cases due to past history of multiple surgeries of the abdominal wall. Least free reported free surgical margin was 3 mm in a 33 years old female with 38 months follow up after excision, but 21 cases were excised with more than 10 mm free margin reaching up to 30 mm as a maximum recorded free margin. No any recorded cases for recurrence after surgery.

**Table 1:** Characteristics of the studies patients with abdominal wall mass (AWE).

Characteristics	Values
Mean age (yr)	35 ± 7.7
Mean of mass size (mm)	42 ± 7
Mean period from CS to first presentation of AWE (months)	55.2 ± 25.3
Mean Hemoglobin level (grams per deciliter)	10.9 ± 2
Main patient complaint	total number (n = 30)
cyclic pain	n = 19
mass	n = 7
a symptomatic	n = 4
Surgical procedure	total number (n = 30)
WLE without mesh	n = 28
WLE with mesh	n = 2
Operative department	total number (n = 30)
General surgery	n = 21
Gynecology	n = 9
Free surgical margins	total number (n = 30)
Below 1 cm	9
More than 1 cm	21
Preoperative FNA cytology	total number (n = 30)
Yes	n = 3
No	n = 27
Post operative significant complications	total number (n = 30)
Infection	n = 5
Bleeding	n = 4
Hernia	n = 1
No	n = 20
Recurrence	Zero cases

**Fig. 1:**
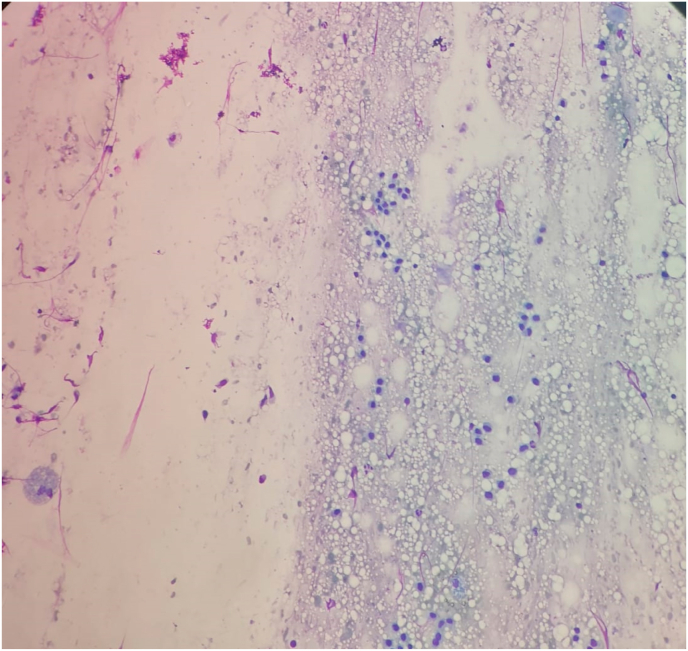
Cytology photomicrography of a case of AWE showing few epithelial cells, scattered stromal cells and hemosiderin laden macrophages (Geimsa stain, 200x).

**Fig. 2:**
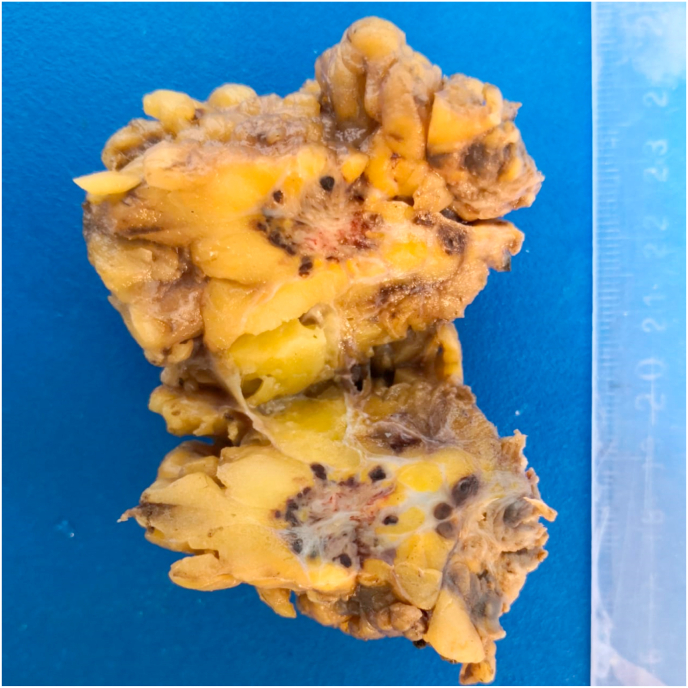
A gross picture of a case of AWE specimen; cut surface showing adipose tissue infiltrated by a defined grey-brown mass with small cysts. . (For interpretation of the references to colour in this figure legend, the reader is referred to the Web version of this article.)

**Fig. 3:**
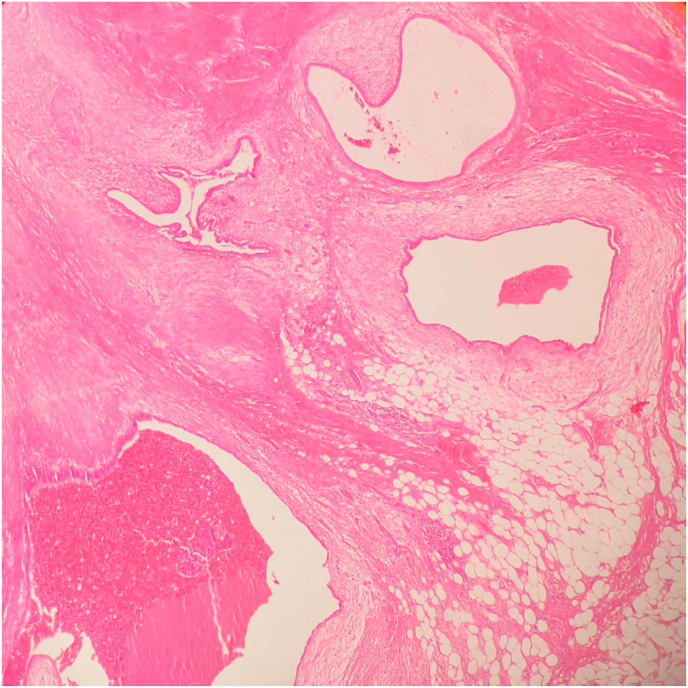
A histopathology section of a case of AWE showing endometrial glands, stroma and occasional hemosiderin in fibrocollagenous and adipose tissue background (Hematoxylin and Eosin staining, 40x).

In view of the pathology examination, preoperative Fine Needle Aspiration Cytology (FNAC) was performed for three cases to exclude granuloma, desmoplasia and malignancy, all three cases showed non specific cytological features, not compatible with tumors or granuloma (Fig. 1). Macroscopic features of the excised masses revealed fairly defined or well defined firm grayish brown areas with occasional certifications suggesting endometriosis (Fig. 2). Histopathological examination showed endometrial glands and stroma within fibrocollagenous scar tissue and adipose tissue with occasional hemosiderin and macrophages (Fig. 3).

The clinical–pathological agreement value for detection of the nature of the abdominal wall mass was 93.4%. The applied Interrater reliability Cohen's kappa coefficient (κ) showed almost perfect agreement (0.9).

## 4 Discussion

Endometrial tissue has been recorded in numerous surgery-related scars, as well as skin, subcutaneous tissues and abdominal wall muscle adjacent to the scars, which forms mass lesion referred to as AWE or scar endometriosis [13,14]. Differential diagnosis of an anterior abdominal wall mass in women of reproductive age usually includes scar endometriosis which is a rare occurrence resulting from pelvic surgical intervention, most commonly CS operations [15]. Various theories have been mentioned to explain the etiopathogenesis of AWE. One theory (transport theory) explains that direct inoculation and the transport of the endometrial tissue into a surgical scar or its adjacent tissue during surgery is responsible for AWE. Another theory (metaplastic theory) suggests that the primitive pleuropotential mesenchymal cells which have undergone metaplasia and differentiation which lead to the development of AWE [16].

The CS is the most frequently performed surgical operation worldwide. Over the past few decades, the rate of CS delivery has been rising rapidly [17]. The long standing WHO advice of 10–15% of deliveries by CS, but this percent is exceeded in many high-income places (average rate of 27%) and low to middle-income settings (up to 29%) [18,19]. In Egypt, The number of birth deliveries in the private sector is increasing and appears to be associated with making the proportion of CS deliveries in 2014 quadrupled the maximum recommended threshold for CS by the WHO [7]. It is well known that the prevalence of CS-associated complications increases with each additional CS operation and these include AWE [20]. In this study at our tertiary hospital, 0.21% of CS cases were complicated by AWE over minimum 12 months and maximum 118 months following CS. The extra-genital endometriosis disease accounts for 6% of all external endometriosis patients and AWE accounts for 0.4%–2.0% of all patients diagnosed with endometriosis [21,22]. But the AWE condition following CS has an estimated incidence of 0.07%–0.47% [23]. Our percent is consistent with Marras et al. reporting an estimated incidence of AWE after CS was 0.23% [24].

The time from CS to the onset of AWE symptoms considerably varies in the literature, ranging from few months to 17.5 years, with an average of 30 months [[25], [26], [27]]. In our study the mean time was 55.2 months which is nearly duplication of this reported mean time in many previous studies, we suggest inaccurate diagnosis or patient's neglecting of a symptomatic abdominal wall masses leads to this late onset, however, this time period comes in agreement with a study performed in South Korea by Song et al. which reported mean period between previous CS and AWE onset of 5.93 ± 4.29 years [22].

The mean age of patients in this study was 35 years ranged between 25 and 55 years, which is consistent with the study done by Marras et al., in 2019 [24] recording mean age of 36 and the Malutan et al. study which reported mean age of 34 years, but slightly lower than the recorded mean age in Song et al. study in 2020 [22], Karaman et al., in 2014 reported a case of 24 years old [21] and Teng et al., in 2008 reported 22 patients of AWE between the ages of 26 and 43 years [27]. Van Langendonckt et al. reported high levels of hemoglobin in the peritoneal fluid of patients with external endometriosis suggesting that heme may be involved in development or pathogenesis of endometriosis [28]. We reported a mean blood hemoglobin level of 10.9 ± 2, with a range between 7.5 and 14.7 (grams per deciliter) with no reported clinical significance.

The mean mass size for the largest diameter was 42 ± 7 mm which is much higher than the mean mass size of AWE cases studied by Marras et al. which was 24 ± 12 mm [24], and Song et al. who reported mean size 32 ± 12 mm [22]. This increase in the mean mass size in our studied cases becomes in relation with the prolonged time for the first onset detection. The main symptoms at presentation in our study included cyclic pain in 19 patients (63.3%) followed by feeling of mass lesion by 7 patients (23.3%), however 4 patients were asymptomatic and accidently discovered during a medical examination. Accidently discovered unusual findings are not uncommon and reported in many sites including the abdominal organs, skin and subcutaneous tissues [2,29,[30], [31]]. Teng et al. [26], found that all patients but two in their study had different severity of cyclic pain associated with menses saying that the diagnosis of AWE appears to be difficult if cyclical pain is not present. Its clinical diagnosis is confused with desmoid tumor, suture granuloma, abscess, hematoma, sarcoma, and metastatic malignancy [32,33].

It is challenging to find a highly sensitive and specific preoperative diagnostic tool for endometriosis as the etiology and pathophysiology is still not fully understood.A definitive diagnosis can be made by histopathological evaluation after biopsy or excision, preoperative ultrasonography, computed tomography (CT), magnetic resonance imaging (MRI) are less valuable in the diagnosis of endometriosis, [21]. A study was done by Ribeiro Júnior et al. to determine the frequency of p53 codon 72 polymorphism in Brazilian patients with endometriosis revealing that the p53 polymorphism can be used as a promising molecular marker for symptomatic endometriosis, and therefore could be a great aid in the diagnosis, guiding prognosis, and treatment of external endometriosis [34]. Circulating Endometrial Cells (CECs) are also detected in most patients with histopathologically proven endometriosis. Early detection of CECs in the peripheral blood of women with pelvic pain or post-CS abdominal wall mass, in addition to objective clinical examination suspecting endometriosis lesion, could accelerate and improve diagnosis.

The gene expression profiling of endometriotic lesions and the parallel CECs samples from peripheral blood identified a range of potential biomarkers including the elevated gene expression of *NANOG, KRT18,* and *VIM* or *of KRT19* and *ESR1* that may be used to identify CECs in the patients with undiagnosed endometriosis [35].

Similarly, circulating stromal cells, CD10^+^ cells, in the circulating blood of endometriosis women were detected using size-based separation approach (ScreenCell®) [36].

FNAC can be a useful tool in diagnosis of cutaneous and subcutaneous endometriotic lesions, providing a rapid and accurate preoperative diagnosis, particularly for differentiation from the metastatic disease, lipoma, cysts and desmoids tumor [33]. Cytological examination of the smears from AWE shows varying cellularity comprising both epithelial and spindle stromal cells, scattered inflammatory cells and variable number of hemosiderin laden macrophages, the presence of any two of the main three components of endometriosis (endometrial glands, stroma cells and hemosiderin laden macrophages) has been agreed for the cytological diagnosis of external endometriosis in both cytology and histopathology, but needle puncture of subtle endometriotic lesions may promote their progression and development. Therefore, the AWE should not be traumatized when possible [37,38]. It is worth to be mentioned that the cytology features of AWE are related to the cyclical hormonal changes; In proliferative phase, the epithelial endometrial cells form cohesive sheets of small uniform cells with scant cytoplasm and round to oval nuclei with bland chromatin and occasional mitosis. In the secretory phase, the size of the cell gradually increases showing cytoplasmic micro vacuolations and the stromal cells show abundant cytoplasm with pre-decidual change, causing diagnostic difficulties. The background looks sanguineous with some inflammatory cells and histiocytes ± hemosiderin [33]. Epithelial cells in secretory phase may show squamous, tubal or mucinous metaplasia with nuclear atypia, so the reporting cytopathologist should be aware of this for accurate diagnosis and differentiating benign and malignant abdominal wall masses [39]. Only three cases in our study were advised for preoperative FNAC due to suspicious tumors and all the three cases were suggestive for endometrioma, the history of CS in all the studied cases made the clinical suspicion for AWE not very difficult that made the Kappa value indicating almost perfect agreement between the clinic-radiological features and the pathology final result. No recurrence or malignant transformation was recorded in our study. The unexpected malignant transformation of benign lesions was recorded in several lesions including the AWE, where Song et al. found three malignant cases out of 38 AWEs (7.9%) [22,40,41].

Some authors have recommended initiating medical therapy for external endometriosis, such as gonadotropin-releasing hormone and oral contraceptives to avoid recurrence after surgery [29]. We did not find complete data about using the non-surgical modalities for the studied cases or other AWE medically treated patients at our hospital, so we cannot support this recommendation. However it is a feasible option for women close to menopause, but it is not an effective AWE primary treatment for most of cases [24]. Aromatase inhibitors can be adopted as a non surgical therapy when dealing with large abdominal wall endometriomas since wide local excision could result in losing a considerable of the abdominal wall or can be used as a second-line therapy for patients who are refractory to the standard treatments [42]. Also ultrasound guided percutaneous cryoablation technique seems to be promising, but the surgical excision stills the mainstay of treatment [43]. Wide surgical excision (WSE) with at least a 1 cm margin with patch grafting of the defect is considered a treatment of choice for AWE according to several studies [44]. In this study even the patients who had a surgical margin less than 1 cm (30% of the total cases) did not experience any recurrence. When the aponeurosis is involved, mass excision might need to be followed by closure of the wall using a mesh to lessen tissue tension [43]. In our study two cases had a closure with mesh; one of them revealed incisional hernia and the other had a weak abdominal wall. These two patients underwent parietal repair with a polypropylene mesh placed in a retromuscular position (15–20 cm). However, no available prospective studies on the subject of surgical behaviors associated with increasing AWE risk, minimizing contact of swabs used for cleaning the endometrial cavity within the scar site, removing them quickly from the operation area and avoiding use of the same suture material that was used for closure of the uterus in order to use it to suture the scar site thoroughly saline washing the scar site before closing it may assist to prevent the growth of endometriotic tissue from the scar tissue [45]. Limitation of the study: The overall number of scar endometriosis cases is low due to exclusion of other cases which had surgeries other than caesarian section. Wicherek et al. studied the obstetrical history of eighty one women presenting with AWE after CS and concluded that CS operations performed before spontaneous onset of the labor was associated with an increase of subsequent endometriosis risk [46]. They supposed that the high immune tolerance before the labor onset permitted endometrial cell implantation. We were unable (in this study) to collect the data concerning CS indications.

## 5 Conclusion

AWE with high suspicion of another serious pathology should undergo preoperative cytological biopsy to exclude alternative diagnosis, but this procedure might exacerbate lesions progression, so good correlation between the surgical history and the ultrasonographic findings is crucial and satisfactory for pre-operative diagnosis. Post-operative histopathology evaluation is essential. . Wide surgical excision with margin of less than 1 cm could be accepted especially in case of weak abdominal wall. More studies on the post-CS complications; risks, prevention, early detection and proper management should be encouraged.

## Provenance and peer review

Not commissioned, externally peer-reviewed.

## Funding

This study did not receive any funding from governmental or private organizations.

## Ethical approval

Local Ethical approval was obtained.

## Consent

Electronic written informed consent was obtained for publication of this study.

## Author contribution

Study concept or design: AH, AD, KM, MN, DMT.

Data collection: AH, AD, NO, KM, ZF, AY.

Data interpretation: AH, NO, MN, AAR, DMT.

Literature review: AH, ZF, AS, MAM, AAR, DMT.

Data analysis: AH, MAM, AY.

Drafting of the paper: ALL.

Editing of the paper: ALL.

Manuscript revision: ALL.

## Registration of research studies

ClinicalTrials.gov Identifier: NCT04639063.

## Guarantor

Dr. Abdulkarim Hasan.

## Declaration of competing interest

The authors declare no conflict of interest.
